# Beyond Acute Pain: Understanding Chronic Pain in Infancy

**DOI:** 10.3390/children3040026

**Published:** 2016-11-09

**Authors:** Miranda DiLorenzo, Rebecca Pillai Riddell, Liisa Holsti

**Affiliations:** 1 Department of Psychology, York University, Toronto, ON, M3J 1P3, Canada; mgdilo@yorku.ca; 2 Department of Psychiatry, The Hospital for Sick Children and the Department of Psychiatry, University of Toronto, Toronto, ON, M5T 1R8, Canada; 3 Department of Occupational Science and Occupational Therapy, B.C. Children’s Hospital Research, Vancouver, BC, V5Z 4H4, Canada; liisa.holsti@ubc.ca; 4 Women’s Health Research Institute, Vancouver, BC V6H 3N1, Canada

**Keywords:** infant, pain, acute, chronic, NICU, persistent pain

## Abstract

This topical review presents the current challenges in defining chronic pain in infants, summarizes evidence from animal and human infant studies regarding the biological processes necessary for chronic pain signaling, and presents observational/experiential evidence from clinical experts. A literature search of four databases (CINAHL, EMBASE, PsycINFO, and MEDLINE) was conducted, along with hand searches of reference lists. Evidence from animal studies suggest that important neurophysiological mechanisms, such as the availability of key neurotransmitters needed for maintenance of chronic pain, may be immature or absent in the developing neonate. In some cases, human infants may be significantly less likely to develop chronic pain. However, evidence also points to altered pain perception, such as allodynia and hyperalgesia, with significant injury. Moreover, clinicians and parents in pediatric intensive care settings describe groups of infants with altered behavioral responses to repeated or prolonged painful stimuli, yet agreement on a working definition of chronic pain in infancy remains elusive. While our understanding of infant chronic pain is still in the rudimentary stages, a promising avenue for the future assessment of chronic pain in infancy would be to develop a clinical tool that uses both neurophysiological approaches and clinical perceptions already presented in the literature.

## 1. Introduction

Pain is defined as a negatively-valenced experience with core sensory, social, emotional, and cognitive properties [1]. Unfortunately, due to the sole reliance on self-reporting, infants were initially thought to be incapable of experiencing pain due to their inability to use language to communicate their subjective experience. The seminal work of pioneering scientists [2,3] combined with a public outcry from parents [4], helped end a troubling era of medical practice where scientists and clinicians generally did not acknowledge infant pain [5]. Indeed, research using both animal and human models provides strong evidence to show that infants have the necessary peripheral and central anatomical and neurophysiological systems required for nociceptive transmission, even at very early gestational ages [6,7,8]. Moreover, in humans, unexpected early pain and stress exposure is associated with long-term changes in brain structure (e.g., reduced white matter microstructure and subcortical grey matter), pain processing (e.g., dorsal horn central desensitization), stress-response system functioning (e.g., high basal cortisol levels), and development (e.g., poorer cognition and motor function), particularly for preterm infants cared for in neonatal intensive care units (NICUs) [9,10,11,12]. In response to this growing body of evidence, a plethora of measures have been developed to assess acute infant pain [13,14], and various trials have been conducted that evaluate the effectiveness of pharmacologic [15], behavioral, and physical treatments aimed at mitigating the adverse effects of acute procedural and post-operative pain in infants [16,17,18].

Most recently, both in the clinical setting and in the research community, more attention has been directed at understanding and investigating pain that persists beyond the acute period in infants [19,20]. Currently, no uniform definition exists for chronic pain of infants that has received widespread endorsement. The International Association for the Study of Pain defined chronic pain in adults as pain that lasts or recurs for more than 3 to 6 months after an injury [21]. However, the applicability of using an arbitrary timeline to define chronic pain for infants who have not lived long enough, yet who have long-lasting (relative to their age) painful conditions, such as osteogenesis imperfecta or epidermis bullosa, appears inappropriate [19]. Thus, it is clear that a specific time criterion cannot be applied specifically to newborn infants. The terms “chronic” or “persistent” pain (these terms are used both interchangeably and differentially in the literature) will both be applied in this paper as used by the original authors or best fitting the context deemed by the current authors. Both these labels (chronic or persistent) have also been defined as pain that has no biological value, pain that persists beyond normal healing time (i.e., ‘non-functional’, with acute pain being considered ‘functional’), or pain that persists when repair has seemingly ended [21]. However, these definitions do not take into account basic physiological mechanisms of this condition, which does not provide clarity regarding treatment and prevention. Instead, a definition is needed with greater focus on models that define infant chronic pain based on the underlying mechanisms of the nervous system. Given that the generalizability of such neurobiological animal models to explain pain in adults has yet to be applied definitively, the application of neonate animal models to the unique context of the developing infant nervous system appears premature.

Thus, for this topical review, the foci will be to: (1) summarize the evidence from animal and human infant studies reporting on the neurophysiological processes that underlie different types of chronic pain in infancy; (2) present evidence gathered from expert researchers and clinicians about infant chronic pain. Our intention is to integrate these lines of evidence, in order to further our understanding of pain beyond acute in infancy, and to inform future research and clinical recommendations.

## 2. Materials and Methods

The literature search considered peer-reviewed papers included in the following electronic databases: the OVIDSP platform was used to run the search strategy in MEDLINE and EMBASE, ProQuest was used for PsycINFO, and EBSCOHost was used for CINAHL. Articles indexed from inception to 12 November 2012 were included in the initial search and the search was updated in August 2016. Electronic search terms included “infant newborn”, “infant premature”, “chronic pain”, “nociceptive pain”, “intractable pain”, “ex-prematurity”. To be included in this review, papers had to: be written in the English language, have an abstract available online, and involve infant pain that persists beyond the acute period. The search yielded 262 abstracts that were independently assessed against the above pre-specified inclusion criteria. Additional literature was gathered by reviewing the reference lists of the papers obtained from the original search. The literature was sorted into categories relating to neurophysiological processes that focused on pain beyond acute, and clinical studies that highlighted the applied aspects of pain beyond acute in infancy. In total, 58 papers met the criteria for inclusion in the current review.

## 3. Neurophysiological Developmental Changes in Pain Processing

Compared to temporal or evaluative definitions, current conceptualizations outside of infancy assert that biological mechanisms underlying the pain state are the best way to categorize chronic pain. For example, Woolf [22] proposed a three-pronged classification involving nociceptive pain that provides early signaling of damage, and inflammatory pain which inhibits movement to promote healing. Both nociceptive and inflammatory pain are adaptive and protective. In contrast, “pathological” pain, which can be further divided into neuropathic pain (involving nerve damage) and dysfunctional pain (reflecting abnormal nervous system function) is maladaptive and is considered a “disease of the nervous system”. It is clear that a neurophysiological approach to defining chronic pain in infancy is necessary; however, understanding the underlying mechanisms has provided a challenge for researchers because of the immature infant nervous system.

While much of the pain processing system in adults is also functional in infants, the first year of life is marked by significant structural and functional changes in pain pathways, and as a result, pain is processed differently in infants than in adults [23,24]. Animal models, particularly those using rats, tell us a lot about the developmental changes that occur in infant pain pathways, since the changes seen in the two post-natal weeks of rats parallel the neurobiological development in human infancy [25]. This research using animal models has helped to highlight certain developmental changes in somatosensory circuitry that occur over the period of infancy, which have direct relevance to the processing of persistent or chronic pain states in infancy.

Generally, researchers have noted early maturation of nociceptive processing in the periphery of neonate rats; however, their central processing of pain develops more slowly [25]. Early in development, large diameter A*β* primary afferent fibers, which typically relay non-noxious tactile information to the dorsal horn, extend beyond their final resting place in the dorsal horn during infancy [26,27]. The functional implications of the immature A*β* fiber termination is that the low threshold fibers are able to access and activate high-threshold Aδ and C-fibers that usually process noxious information [26]. Consequently, as shown in a study evaluating premature human infants, infants are unable to discriminate between non-noxious and noxious inputs until approximately 35 weeks’ gestational age [6]. Further research in neonatal rat models shows that the low threshold tactile afferents (A*β* fibers) retract from the site of high-threshold input until completely segregated after a few weeks of life [28].

In addition to the structural changes of primary afferent innervation of the dorsal horn in early life, key developmental changes also occur in the intrinsic properties of the dorsal horn during infancy. Using rodent models, at birth, the receptive fields in the dorsal horn are large, and decrease in size over the first two postnatal weeks. Stimulation of these “wider” cutaneous receptive fields at early postnatal ages can enhance signaling and evoke long-lasting excitation [29,30]. Furthermore, many neurotransmitters and signaling molecules involved with nociceptive processing are expressed early in development in rodent models, but do not reach adult levels until after infancy [25,31]. Particularly, an imbalance exists between excitatory (e.g., glutamate) and inhibitory (e.g., GABA and glycine) neurotransmitters and their respective receptors that favors excitatory transmission [25]. An increase in excitatory input is observed because both glutamergic receptors, *N*-methyl-d-aspartate (NMDA) receptor and α-amino-3-hydroxy-5-methyl-4-isoxazolepropionic acid (AMPA) receptor, are highly expressed in the neonatal rat spinal cord before being down-regulated to adult levels [32,33,34]. Further contributing to this imbalance is the fact that particular inhibitory mechanisms are either weak or absent in early postnatal life. Indeed, glycinergic mini-inhibitory postsynaptic currents are absent within the most superficial laminae of the dorsal horn until the second postnatal week of rodent models, but responses to exogenous glycine can still be evoked [35]. These research findings suggest that during infancy the functional receptors of glycine may be present, but that the glycinergic neurons are absent, resulting in less inhibitory signaling. Additionally, GABA has a reduced inhibitory drive at birth compared to adulthood. A balance of spinal excitability and inhibitory mechanisms is imperative for normal tactile and nociceptive processing [28]; however, stasis is not established between the excitatory and inhibitory drives until later in infancy.

A final key neurodevelopmental change in nociceptive processing includes the delayed maturation of brainstem descending pain pathways that play a major role in the control of pain transmission [36]. Descending pathways from the brainstem to the spinal cord are present in utero in animal models, but functional inhibition of nociceptive signals is not thought to be effective until 2–3 weeks post-delivery [37,38]. Hathway et al. [39] showed that in rats, the rostroventral medulla (RVM), which houses the main output nuclei for brainstem descending control, undergoes drastic maturation after the 21st postnatal day (P21). Lesioning and electrical stimulation of the RVM has revealed that its control over nociceptive circuits is entirely facilitatory before P21 in rats [39,40]. It is only after three postnatal weeks that inhibitory pathways exert an influence. Researchers have argued that the RVM-mediated facilitation of nociception in early life plays a significant role in sustaining pain from neuropathic and inflammatory injury [39,41]. Importantly, the functional development of the descending inhibitory pathways has been shown to be concomitant with the refinement of noxious withdrawal reflexes observed in early human development [42]. Therefore, together, these significant neurobiological developmental changes suggest that infants are more, rather than less, sensitive to pain than older individuals.

## 4. Types of Chronic Pain in Infancy

Neuropathic pain and inflammatory pain are the two main types of chronic pain [43]. Inflammatory pain manifests as a consequence of tissue damage/inflammation (e.g., surgical injury with resulting tissue inflammation) and neuropathic pain arises from nervous system lesions (e.g., brachial plexus injuries). Neuropathic pain is rare in infants; whereas, persistent inflammatory pain is observed more frequently [31]. The development of inflammatory pain, or the protection against the development of neuropathic pain, can be ascribed to certain pain circuitry immaturities, such as those discussed previously.

### 4.1. The Development of Persistent Pain Associated with Tissue Injury and Inflammation

The development of an inflammatory pain syndrome in response to tissue injury has been well documented in animal models [24]. Plantar hindpaw incision is a post-operative pain model used widely as it is highly reproducible in both neonatal and in adult rodents [31]. In neonatal rodents, surgical incisions of the plantar hindpaw produce inflammation-induced primary hyperalgesia (decreased pain threshold) from the age of P3 to P17 [44]. Hyperalgesic responses to injury in neonates is not specific to surgical trauma. Inflammogenic molecules, such as mustard oil, can also cause hyperalgesic responses in neonatal rats. Mustard oil is a potent activator of C-fibers (nociceptive cells) and produces primary hyperalgesia and secondary hyperalgesia (pain arising from the tissue surrounding a wound) in both adult and in neonatal rats [45]. However, the pain responses to mustard oil are smaller in neonate rats compared to adult models, which reflects neurodevelopmental immaturities of nociceptive cells within the dorsal horn [46].

More long-term consequences of inflammation associated with hindpaw incisions in neonatal rat models, include significant changes in the resting membrane potential of large diameter sensory neurons that persists beyond the time in which behavioral pain responses are observed [47]. The change in resting membrane potential causes the sensory neurons to be much more excitable. Aforementioned neurobiological immaturities, such as the relative lack of brainstem inhibitory descending controls and the imbalance of inhibitory and excitatory neurotransmitter activity in neonates, are purported to play a role in this biophysical change observed in resting membrane potential [31]. Another example of long-lasting consequences of tissue injury involves changes in microglia, which are cells within the central nervous system that contribute to the manifestation of chronic pain states in animal models [43]. Neonatal skin incision changes the phenotype of microglia, which can alter sensory afferent cells, intrinsic dorsal horn cell responses, and consequently, cause an increase in sensory neuron excitation.

In comparison to rat models, direct evidence of the development of pain associated with inflammation and tissue injury from human infant studies is quite limited. Follow-up studies of infants hospitalized in the NICU, or those who had surgery during the neonatal period, show that they are vulnerable to aspects of altered central pain processing which appear to place them at heightened risk for developing chronic pain. For example, following exposure to heel lance and to surgery with anesthesia, infants had lowered thresholds to tactile stimulation up to the post conceptual age of 35 weeks (sensitization) [48] and decreased thresholds during the first year of life (primary hyperalgesia) [49]. Secondary hyperalgesia was also exhibited in infants, even when the injury was to the contralateral side [50]. In addition to secondary hyperalgesia, infants develop allodynia (pain arising from previously innocuous stimulation) as a result of central sensitization [3,51].

What is not clear is whether or not these changes observed in human infants could be considered “adaptive”; that is, sensitization after minor and major trauma is meant to be protective [52]. As such, the question arises whether evidence of these neonatal changes in pain processing systems are preliminary evidence for chronic pain and thereby considered “maladaptive” [22]. Interestingly, even though the “priming towards excitation” described above suggests that these infants could be at a heightened risk for developing chronic pain because of maladaptive processes, another line of research indicates that infants may be “protected” from some forms of chronic pain [53].

### 4.2. The Development of Pain Associated with Nerve Injury

Research in rodent models does suggest protection against the development of chronic neuropathic pain in infancy [54,55,56,57,58]. After peripheral nerve injury, persistent mechanical allodynia does not occur until 3 post-natal weeks [54]. In addition, microglia in the dorsal horn involved in the immune response to nerve injury are activated only at very low levels following nerve injury in young animals even up until P16 [55]. This response does not occur because neonatal animals lack innate immune responses, but is more likely a result of absent T-cell activation and infiltration indicating that nerve damage has taken place [56]. Thus, since infant rats are capable of developing clear pain hypersensitivity upon inflammation [44], the lack of neuropathic pain behavior following nerve injury in young animal models has been attributed to immature neuroimmune pathways, rather than a failure of pain processing [57]. In line with this notion, a more recent study by McKelvey et al. offers a novel mechanistic explanation for the absence of pain following nerve damage in infancy. In contrast to adult nerve injury, which triggers a proinflammatory immune response in the dorsal horn, nerve injury in infants triggers an anti-inflammatory immune response with significant increases in IL-4 and IL-10 [58]. Interestingly, the blockade of the anti-inflammatory activity of IL-10 unmasks neuropathic pain behavior suggesting that nerve injury in infancy is not absent, but rather is suppressed by the IL cytokine proteins. Nevertheless, the immature presentation of these factors associated with the development of pain associated with nerve injury have not been studied in human infants and thus provides exciting lines of evidence to pursue.

Similar to the neuropathic pain animal models, under some conditions in which adults and older children would be at risk for developing neuropathic pain, infants appear to be protected. For example, infants who experience complete avulsion of the brachial nerve at delivery, when assessed between 3 and 23 years, do not report chronic pain; and in fact, following surgical repair, they show vastly improved sensory recovery compared to motor recovery, and have normal sensation in other areas of the body [59]. Very few infants with this injury go on to exhibit behaviors which may indicate ongoing chronic pain in childhood [60]. Some postulate that these results occur because nerve conduction velocity in children under the age of two years is 50% lower than in adults [59,61]. In addition, very few children with congenital amputations report phantom pain (<10%) [62]. Yet, in older children (5–19 years) and in adults who undergo amputation, prevalence rates of phantom pain can be as high as 80% [63,64]. Moreover, associations between developing persistent pain after inguinal hernia repair were very low in those operated on in the first 3 months of life [65]. Indeed, Tsai et al. found that infant postoperative chronic pain was minimal and transient after inguinal hernia repair [66]. A similar trend was found for thoracotomy and strenotomy procedures; the prevalence of chronic pain was low in children that underwent these procedures before school entry [67,68].

## 5. Reconciling Clinical Observations with Pre-Clinical Research

While biologically-based models, particularly rodent models, provide an important foundation for building a conceptualization of the mechanisms of infant persistent pain, actual clinical experience with infants suspected of having ongoing pain provides important complementary information.

To explore the perspectives of infant caregivers on “chronic pain” in infants, based on their observations and caregiving experiences, Pillai Riddell et al. conducted in-depth interviews with a multi-disciplinary sample of highly experienced clinicians (average years of experience was 17 years) from three separate university-affiliated tertiary care centers [19]. While most accepted the idea of chronic pain existing in infants, agreement in regards to a definition was elusive. In contrast to the adult literature where chronic pain is most often cited as pain lasting more than 3 to 6 months [69], a significant proportion of clinicians thought that infants could experience persistent pain after weeks if the context included a clinical presentation of a disease type known to be associated with ongoing pain in infants, such as in epidermolysis bullosa.

Pillai Riddell et al. highlighted another definitional issue on pain beyond acute that was expressed by clinical experts identified as ‘iatrogenically prolonged’ pain, which is applicable to infants requiring prolonged hospitalization in the NICU [19]. They differentiated infants with unremitting conditions that cause persistent pain, from those undergoing a series of acute or acute-prolonged pain experiences (i.e., pain induced and maintained by repetitive medical procedures) that result in a “chronically pained state”. Thus, the acknowledgment was made that these infants may not have a persistent pain state resulting from changes in the nervous system, but rather experience prolonged pain as a result of iatrogenic procedures that are added cumulatively to pain related, earlier procedures that have yet to abate.

Both a sample of clinicians and a follow-up study with parents who had infants currently suspected of having chronic pain (conservatively defined as conditions known to be chronically painful in older populations) independently described two types of infant persistent pain behavioral profiles: the “hyperreactive” and “hyporeactive” infant [19]. The majority of health professionals in these samples suggested that an infant who is “hyporeactive” (exhibits little to no reaction to an acute pain procedure), may be experiencing chronic pain. However, other professionals in the sample suggested the opposite, whereby an infant who is “hyperreactive” (exhibits an exaggerated response to an acute pain procedure), indicates that an infant is experiencing chronic pain. These differing labels further confound the problem of how best to define chronic pain in infants, and consequently, how to integrate the aforementioned findings from pre-clinical and clinical science. The “hyporeactive” infant does not appear to interact with caregivers or even to react to extensive noxious stimulation. Health professionals describe infants as “hyporeactive” if they do not respond behaviorally when a known painful procedure is performed. In contrast, the “hyperreactive” infant exhibits heightened distress even in anticipation of an impending noxious procedure and was described to have behaviorally higher peak distress during painful procedures.

More recently, using a web-based survey technique, van Ganzewinkel et al. invited international neonatal experts and parents to define chronic pain [20]. When responding to the questions relating to what is chronic pain, experts described no known endpoint and they described a concept whereby pain is present, despite the lack of a proximal event or procedure. In addition, consensus by the group indicated that pain would interfere with development and prolong healing times. Not surprisingly, when etiology was discussed, rather than biological mechanisms relating to aberrant pain pathways, painful conditions and situations were listed. However, specifics with regards to diagnostic factors could not be identified.

## 6. A Need for Chronic Pain Assessment

Both the aforementioned studies working with clinicians suggest that the manifestation of pain beyond acute in infancy is still not well understood. Encouragingly, despite the lack of definitive criteria regarding the mechanisms or the definition of chronic pain in infancy, the belief that it does exist appears to be commonly accepted. Reaching a consensus on both clinical observations and neurobiological mechanisms of chronic pain in infancy is necessary to develop valid assessments. Currently, neither clinicians nor researchers have yet to devise a strategy that can definitively determine whether or not a newborn is experiencing chronic pain, and as a result, chronic pain is difficult to treat effectively [20]. Although a plethora of assessments for acute pain exist, with varying psychometric properties established, most research to date indicates that behavioral and physiological markers of acute pain may not be applicable to chronic pain [70,71,72]. Researchers have suggested that clinical observations in the NICU (e.g., NEOPAIN trial) [73] and the development of scales, such as the COMFORTneo scale [74] or the EDIN scale [75], show promise in isolating indicators that do not focus on simply peak distress response [74]. Nevertheless, pediatric pain experts have yet to agree on any type of clinical cluster of behaviors or physiological indicators that could form the basis of the tool. The lack of self-reporting in infancy further complicates these problems. Given that infants who have been exposed to prolonged pain have been described as both over and under-responsive, clinicians are unsure of how to determine whether or not the unresponsive infant has stopped responding because of the cessation of pain or has exceeded its capacity to respond to too much pain.

## 7. Concluding Remarks

Despite decades of research demonstrating that human infants are capable of experiencing pain, the evidence between acute and chronic pain and between animal and human research has developed disproportionately. This has resulted in limited knowledge of how the pre-clinical science on chronic pain relates to the human infant experience, although an understanding of acute behavioral responses to neonatal and infant pain appears generally well-established. However, the potential for infants’ capacity for chronic pain is still in need of fundamental research across different pain paradigms (e.g., postsurgical persistent pain syndromes, iatrogenically-prolonged pain exposure). From such a foundation, researchers and clinicians can build a knowledge-base of persistent pain during early human life that reconciles what is known from the collective lines of research. Longitudinal prospective studies tracking infants from birth who are suspected of chronic pain conditions may help to rectify the different clinical pictures seen in chronic pain. The ultimate goal is that a clinical tool is eventually designed that effectively marries both the physiological substantiated mechanisms and clinical perceptions that have been already offered in the literature regarding chronic pain in infancy. This convergence of biological and behavioral/observational evidence could ultimately inform a more accurate assessment, and safe, effective, and ethical treatment of infants who suffer from pain beyond the acute period.
